# Factors Associated with Adverse Birth Outcomes in Women with an Intellectual or Other Developmental Disability

**DOI:** 10.3390/healthcare13070780

**Published:** 2025-03-31

**Authors:** Kyle Bradford Jones, Isabel K. Taylor, Tyson Schwab, Camille King, Godwin Okoye, Jaewhan Kim

**Affiliations:** 1 Department of Family and Preventive Medicine, University of Utah Spencer Fox Eccles School of Medicine, 240 Morris Ave. #400, South Salt Lake City, UT 84115, USA; tyson.schwab@hsc.utah.edu; 2 Department of Pediatrics, University of Utah Spencer Fox Eccles School of Medicine, 50 Medical Drive, Salt Lake City, UT 84132, USA; 3 Utah Valley Family Medicine Residency, 475 West 940 North, Provo, UT 84604, USA; camille.allison@imail.org; 4 Health Outcomes Division, College of Pharmacy, The University of Texas at Austin, 2409 University Avenue Stop A1900, Austin, TX 78712, USA; gokoye@utexas.edu; 5 Physical Therapy and Athletic Training, University of Utah College of Health, 520 Wakara Way, Salt Lake City, UT 84108, USA; jaewhan.kim@utah.edu

**Keywords:** IDD, adverse birth outcomes, preterm birth, low birth weights, UPDB

## Abstract

**Objective.** Women with intellectual or developmental disabilities (IDDs) experience poorer prenatal care and worse perinatal health and birth outcomes than the general population. The purpose of this study is to describe the maternal characteristics and to identify factors associated with the increased risk of adverse birth outcomes among women with an IDD. **Methods.** Electronic medical records and the Utah Population Database were used to identify demographic and medical characteristics of pregnant individuals between 14 and 45 years old with an IDD and the related birth outcomes. Random-effects logistic regression was used to identify factors that were associated with adverse birth outcomes. **Results.** A total of 5147 births by 2250 mothers with an IDD (average births per mother = 2.33) were identified. Multigestational pregnancy (twins or triplets) (OR = 32.85, *p* < 0.01), fewer prenatal care visits (OR = 3.01, <0.01), gestational hypertension (OR = 2.74, *p* < 0.01), and the presence of a mental illness (OR = 1.28, *p* = 0.01) had an increased risk for preterm delivery. Associated low birth weight factors included multigestational pregnancy (OR = 22.82, *p* < 0.01), gestational hypertension (OR = 3.23, *p* < 0.01), maternal smoking status (OR = 1.54, *p* < 0.01), fewer prenatal visits (OR = 2.91, *p* < 0.01), and maternal mental health disorder (OR = 1.66, *p* < 0.01). Cesarean deliveries were associated with gestational hypertension (OR = 2.33, *p* < 0.01), Medicaid coverage (OR = 1.76, *p* < 0.01), and gestational diabetes (OR = 1.42, *p* < 0.01). Neonatal intensive care unit (NICU) admission was associated with increasing maternal age, multigestational pregnancy, the number of prenatal care visits, hypertension, and maternal mental disorders. **Conclusions.** These results suggest that sociodemographic factors and health problems put women with an IDD at a higher risk of adverse pregnancy and infant outcomes. Appropriate clinical care and social supports should be utilized to optimize the health and outcomes of this population.

## 1. Introduction

Individuals with intellectual and/or other development disabilities (IDDs) have historically experienced poorer health outcomes, shorter lifespans, and inequities in the healthcare system [1,2]. While the fertility rate of women with an IDD is lower than the fertility rate of women without an IDD (20.3 per 1000 vs. 43.3 per 1000) [3], pregnancy in women with an IDD is increasing over time [4]. Women with an IDD are at increased risk for early labor, preeclampsia, venous thromboembolism, hemorrhage, longer hospital stays, and increased rates of cesarean delivery [5,6,7,8]. These individuals are also more likely to experience emergency department (ED) visits and hospitalizations after giving birth, including for psychiatric conditions, and are more likely to have a second birth within the next twelve months [9,10,11]. Further, the increased rates of cesarean deliveries observed among women with an IDD are not entirely attributable to pre-existing health conditions or pregnancy complications [12].

Compared to women without disabilities, those with an IDD are more likely to experience a range of adverse peripartum conditions, including epilepsy, obesity, mental health issues, and medication use, as well as socioeconomic challenges like poverty [13]. Women with an IDD may also be more likely to delay prenatal care and thus receive fewer prenatal visits; however, this relationship does appear to be dependent on the type of IDD [14].

The previous literature has also suggested that infants born to mothers with an IDD face higher risk of complications including preterm birth, low birth weight, and stillbirth when compared to infants born to women without disabilities [4,6,7,15,16,17]. Further, infants of mothers with an IDD are more likely to be admitted to the neonatal intensive care unit (NICU) [16,18]. Even after controlling for demographic factors, studies have shown higher infant mortality rates for babies born to mothers with an IDD [15,16].

Socioeconomic status (SES) and racial/ethnic disparities, which are often linked to poor health outcomes, may also mediate the relationship between an IDD and adverse birth outcomes. For instance, women with an IDD are more likely to be from lower SES backgrounds and racial/ethnic minority groups, both of which are independently associated with poorer maternal and infant health outcomes [4,19]. Black and Hispanic women with an IDD face higher rates of stillbirth compared to their White counterparts, with Black women also experiencing higher rates of gestational hypertension [4,20]. Studies have also shown that delivering mothers with an IDD are more likely to be from poor neighborhoods and to use public insurance than those without disability [4,13].

Despite the existing research, significant gaps remain in understanding the specific factors during pregnancy that contribute to adverse birth outcomes among women with an IDD. In particular, there has been limited evaluation of this population. The purpose of this study was to describe the maternal characteristics and to identify specific health and demographic factors associated with increased risks of adverse birth outcomes among women with an IDD.

## 2. Methods

**Data and Subjects**. Electronic medical records (EMRs) and the Utah Population Database (UPDB) were used to identify women with an IDD diagnosis who gave birth in Utah between the ages of 14 and 45 years. Birth records from 1996 to 2018 were included as EMRs became available starting in 1996, and 2018 was the most recent year for which Utah birth records were accessible. IDD was identified using International Classification of Diseases-9/10 (ICD-9/10) diagnosis codes from the Centers for Medicare and Medicaid Services (CMS) Chronic Conditions Data Warehouse (CCW) [21]. The CCW defines IDD based on a set of diagnosis codes capturing conditions characterized by significant limitations in intellectual functioning and adaptive behavior. These codes ensured consistency in case identification and facilitated comparability with other studies utilizing CMS data. The developmental disability included intellectual disability (e.g., ICD-9: 317.X, 318.X, and 319.X; ICD-10: F70.X-F79.X), autism spectrum disorder (e.g., ICD-9: 299.X; ICD-10: F84.X), Down syndrome (e.g., ICD-9: 758.X; ICD-10: Q90.9), and cerebral palsy (e.g., ICD-9: 343.X; ICD-10: G80.X).

The EMRs include visit date, age at visit, ICD-9/10 diagnosis codes, current procedural terminology (CPT), and clinical values (e.g., hemoglobin A1C and blood pressure). The UPDB has birth records and socioeconomic status such as education years and marital status. The birth records include all state-wide birth records since 1980 and provide birth date, mother’s age at birth, gestational comorbid conditions, gestational age, prenatal visit, birth weight, any birth complications, and any other anomalies [22].

The UPDB and the EMRs are equipped with unique person identification numbers (IDs) that facilitate linkage. After identifying subjects with IDD in the EMRs using ICD-9/10 diagnosis codes, UPDB programmers linked these subjects to birth files in the UPDB. Because each subject in the UPDB and the EMRs possesses a unique person ID over time, it becomes possible to identify women with multiple births. The birth files in the UPDB encompass all births in Utah during a specific period. All data were anonymized by the UPDB before being obtained by the authors in January 2022. The project was approved by the University of Utah Internal Review Board (IRB_00121259).

**Outcomes.** Outcomes of infants and mothers were identified from the birth records. For the infants, preterm birth, low birth weight, NICU admissions, and infant mortality data within a year from birth were obtained. For the mothers, cesarean delivery and infection were identified. Preterm birth was defined when gestational age was less than 37 weeks, and low birth weight was defined when birth weight was less than 2500 g [23]. Available classification of infections included bacterial vaginosis, chlamydia, gonorrhea, group beta strep, hepatitis C, herpes, human parvovirus, pyelonephritis, syphilis, and urinary tract infection. NICU and infant death within first year of life were identified from the birth records that had an indicator (Yes/No).

**Independent Variables.** Variables that could potentially be associated with the outcomes were identified and controlled. Mother’s age at birth (14–17, 18–25, 26–30, 31–35, and 36–45 years old), mother’s race (non-Hispanic White, Hispanic, and non-Hispanic Other), mother’s education (did not graduate from high school—Yes or No), mother’s marital status at delivery (married—Yes or No), enrolled in Medicaid (Yes or No), smoker (Yes or No) during pregnancy, a multigestational pregnancy (e.g., twins and triplets), and number of prenatal care visits were identified from the birth records. Gestational hypertension (Yes or No), gestational diabetes (Yes or No), obesity (≥30 kg/m^2^ body mass index—Yes or No), seizure (Yes or No), and mental disorders during pregnancy were obtained from the birth records. The mental disorders included depression, anxiety, schizophrenia, and bipolar and were discovered from the birth records and the EMRs. Number of prenatal visits (≤7 visits, 8–15 visits) was categorized and controlled in the regression.

The study aimed to determine which maternal characteristics and health or demographic factors were associated with an increased risk of adverse birth outcomes among women with an IDD. The hypothesis was that specific maternal health and demographic factors (e.g., age, socioeconomic status, prenatal care, and comorbid conditions) would be associated with higher risks of adverse birth outcomes, including preterm birth, low birth weight, NICU admissions, infant mortality, cesarean delivery, and maternal infection.

**Statistical Approach.** Baseline characteristics of the subjects were summarized by mean, standard deviation (SD), and percentage. Because the birth outcomes were binary (Yes/No) and the same subjects could have more than one birth over time, random-effects logistic regression was used to identify factors that were associated with adverse birth outcomes. *p*-values less than 0.05 were considered statistically significant. Stata version 18.0 was utilized for the analysis.

## 3. Results

Of the eligible data from 1996 to 2018, a total of 5608 births by 2451 mothers with an IDD were identified. Exclusions were made for births with missing prenatal care visits (n = 247) and missing education (n = 238), leading to the removal of 201 mothers. The final number of births included in the study was 5147, involving 2250 mothers (average births per mother = 2.33).

The maternal average (SD) age at delivery was 27.0 (5.8) years old, 19% had not completed high school level education, 72% were married at the time of pregnancy, 39% were Hispanic, 9.4% smoked, and 12.6% had Medicaid coverage. Additionally, 7% had gestational hypertension, 4% had gestational diabetes, 11% were considered to have a mental illness diagnosis, and 28% were diagnosed with obesity (Table 1).

About 14% of births were preterm birth, while 11.8% of the infants were born with low birth weight, 24.8% experienced a cesarean delivery, and 8.4% experienced a maternal infection related to delivery. When examining newborn outcomes of infants, 4.8% required NICU care and 1.7% experienced infant death within the first year (Table 2).

Preterm Delivery. The results show an increased risk of premature births among non-Hispanic-Other-identified mothers compared to non-Hispanic White mothers (OR = 1.45, *p* = 0.04). Both mothers with a multigestational pregnancy with twins or triplets (OR = 32.85, *p* < 0.01) and who had fewer number of prenatal visits (OR = 3.01, *p* < 0.01) had a higher association with preterm delivery.

Low Birth Weight. Married mothers were 22% less likely to have a lower birth weight among infants as compared to non-married mothers (OR = 0.78, *p* = 0.03). Additional associations with low birth weight in infants included a multigestational pregnancy (OR = 22.82, *p* < 0.01), maternal smoking during pregnancy (OR = 1.54 *p* < 0.01), fewer prenatal visits (OR = 2.92, *p* < 0.01), gestational hypertension (OR = 3.23, *p* < 0.01), and a maternal mental disorder (OR = 1.66, *p* < 0.01).

Cesarean Delivery. There was an increased occurrence of Cesarean deliveries associated with increasing maternal age (26–30 years old: OR = 1.52, *p* < 0.01; 31–35 years old: OR = 1.77, *p* = 0.01; 36–45 years old: OR = 2.40, *p* < 0.01 as compared to the 18–25-year-old group). Additionally, there was an increased risk of Cesarean deliveries in Hispanic individuals (OR = 1.32, *p* < 0.01), those with a multigestational pregnancy (OR = 6.14, *p* < 0.01), gestational hypertension (OR = 2.33, *p* < 0.01), gestational diabetes (OR = 1.87, *p* < 0.01), and obesity (OR = 1.45, *p* < 0.01).

Infant Outcomes (NICU Admission and Death). This study identified several factors associated with increased NICU admission for infants including increased maternal age (26–30 years old: OR = 1.84, *p* < 0.01; 31–35 years old: OR = 1.71, *p* = 0.01; 36–45 years old: OR = 2.71, *p* < 0.01 as compared to the 18–25-year-old group), a multigestational pregnancy (OR = 7.20, *p* < 0.01), patients with Medicaid coverage (OR = 6.45, *p* < 0.01), mothers who participated in fewer prenatal care visits (OR = 1.68, *p* < 0.01), maternal hypertension (OR = 2.02, *p* < 0.01), maternal diabetes (OR = 1.83, *p* = 0.02), and mothers with a diagnosis of a mental disorder (OR = 1.87, *p* < 0.01). There is an increased risk of infant death within the first year of life associated with a multigestational pregnancy (OR = 2.71, *p* = 0.02) or mothers who participated in 0–7 prenatal care visits (OR = 3.79, *p* < 0.01).

Perinatal Infections. Maternal infection risk associated with pregnancy was associated with increasing maternal age (26–30 years old: OR = 1.79, *p* < 0.01; 31–35 years old: OR = 2.09, *p* = 0.01; 36–45 years old: OR = 2.29, *p* < 0.01 as compared to the 18–25-year-old group), women with Medicaid coverage (OR = 8.07, *p* < 0.01), gestational hypertension (OR = 1.50, *p* = 0.03), and gestational diabetes (OR = 1.62, *p* = 0.02). Mothers with mental disorders had higher perinatal infection risk (OR = 1.98, *p* < 0.01), whereas those with obesity had a decreased association with perinatal infections (OR = 0.76, *p* = 0.04) (Table 3).

In addition to the overall analysis, subgroup analyses (autism, ID, DS, and CP) were conducted to examine potential differences in adverse birth outcomes across disability categories. While the prevalence of preterm birth and low birth weight did not significantly differ among groups (*p* = 0.23 and *p* = 0.62, respectively), other birth outcomes varied significantly across disability types. The logistic regression analysis showed that the results for disability groups, except for the autism subgroup, were consistent with the overall analysis (the result tables for the subgroup analyses were not presented). The differences in the autism subgroup were likely due to its smaller sample size. These findings suggest that while some adverse birth outcomes are comparable across disability categories, others may be influenced by specific disability-related factors, warranting further investigation.

## 4. Discussion

This study aimed to examine the factors associated with adverse birth outcomes among women with an IDD. By analyzing maternal characteristics, pregnancy complications, and infant health outcomes, this study identified key demographic and health-related factors that contribute to the increased risks faced by this population. The results confirm that women with an IDD are at an elevated risk for complications during pregnancy and delivery, as well as adverse outcomes for their infants, in line with the existing literature [5,8,13,15,16]. Our findings highlight the importance of targeted healthcare interventions to mitigate these risks and improve maternal and infant health.

Women with an IDD are at a significantly higher risk for adverse birth outcomes, including preterm birth, low birth weight, and NICU admission [6,7,15,16]. Our analysis identified specific risk factors, such as fewer prenatal care visits, multigestational pregnancies, and maternal smoking, which were strongly associated with preterm birth and low birth weight. Additionally, gestational hypertension emerged as a critical factor contributing to both adverse outcomes, underscoring the need for enhanced monitoring and management of hypertensive disorders in this population. These findings emphasize the necessity of early and consistent prenatal care to mitigate modifiable risk factors and improve pregnancy outcomes, particularly for those at increased risk due to socioeconomic or behavioral factors like smoking.

Our study also confirmed higher rates of cesarean deliveries among women with an IDD, particularly among Hispanic women and those with gestational hypertension, diabetes, or multigestational pregnancies. While higher cesarean rates in women with an IDD have been previously documented [12], our findings suggest that these disparities persist despite medical advancements. Addressing racial and age-related disparities in maternal care is crucial to ensuring equitable healthcare access and improving delivery outcomes for women with an IDD.

Beyond delivery outcomes, infants born to mothers with an IDD face significant health challenges, including increased NICU admissions and higher infant mortality rates. Factors such as maternal age, Medicaid coverage, fewer prenatal visits, and maternal mental health disorders were strongly associated with poor infant outcomes. The observed association between Medicaid status and adverse birth outcomes suggests that socioeconomic disparities play a pivotal role in shaping maternal and infant health. These findings highlight the urgent need for policies that prioritize equitable access to prenatal and postnatal care for socioeconomically disadvantaged women with an IDD.

While SES is a significant contributor to adverse birth outcomes, other physiological, psychological, and systemic factors may also mediate the relationship between an IDD and poor maternal and infant health. Women with an IDD may have underlying health conditions that predispose them to complications during pregnancy. For example, higher rates of obesity, epilepsy, and metabolic disorders among women with an IDD can increase the risk of gestational diabetes, hypertension, and other pregnancy-related complications [24]. These conditions can directly impact fetal development, leading to preterm birth, low birth weight, and other adverse outcomes [25,26]. Additionally, the physical challenges associated with certain developmental disabilities, such as cerebral palsy, may affect the ability to carry a pregnancy to term or increase the likelihood of cesarean delivery [27].

Mental health disorders, which are more prevalent among women with an IDD, can exacerbate the risk of adverse birth outcomes [24]. Conditions such as depression, anxiety, and schizophrenia are associated with poorer adherence to prenatal care, increased stress levels, and unhealthy behaviors like smoking or substance use, all of which can negatively affect pregnancy outcomes [28]. Furthermore, the psychological stress of managing a disability while pregnant may contribute to hormonal imbalances or immune dysregulation, potentially increasing the risk of preterm labor or infections [29]. Women with an IDD often face barriers to accessing timely and appropriate healthcare. These barriers may include communication challenges, the lack of disability-competent care, and stigmatization within the healthcare system [30]. Delayed or inadequate prenatal care can result in undiagnosed or poorly managed conditions, such as hypertension or infections, which can lead to adverse outcomes [30]. Additionally, the lack of tailored interventions for women with an IDD may contribute to suboptimal care during pregnancy and delivery [31].

SES and racial disparities continue to shape health outcomes for women with an IDD. Our results reveal that Hispanic and Black women with an IDD experience higher rates of adverse outcomes, such as gestational hypertension and stillbirth, than their White counterparts, which mirrors findings from prior research [19]. Additionally, women with an IDD from lower SES backgrounds—evidenced by higher Medicaid coverage rates and lower education levels—are at greater risk for negative birth outcomes, suggesting that structural inequities in healthcare access and quality may disproportionately affect this population. These findings highlight the urgent need for policies that address the intersection of disability, SES, and race/ethnicity in maternal healthcare, with a focus on reducing these disparities through equitable access to care.

This study fills a critical gap in the literature by evaluating the specific factors that contribute to adverse birth outcomes in women with an IDD in Utah, a state with a unique demographic profile. Our findings can inform health policies aimed at improving maternal and infant health outcomes by promoting early and consistent prenatal care, addressing socioeconomic disparities, and implementing targeted interventions for women with specific risk factors such as mental health disorders, hypertension, and smoking. Future research should further explore the impact of interventions that promote continuity of care for women with an IDD and investigate the mechanisms underlying the observed racial and socioeconomic disparities.

Despite the strengths of this study, several limitations should be acknowledged. First, the study population primarily consisted of Hispanic and non-Hispanic White individuals, limiting generalizability to other racial and ethnic groups. Second, a more detailed analysis of insurance coverage and healthcare access would provide further insights into barriers faced by this population. Third, certain conditions, such as gestational hypertension, were used as proxy measures for broader complications like preeclampsia; a more granular analysis could enhance understanding of specific pregnancy-related conditions. Additionally, factors such as cohabitation status, rural versus urban residence, and IDD severity were not accounted for in this analysis but could further refine risk assessments. Finally, while multiple pregnancies were associated with increased risks, adjustments for these confounders were not explicitly made, necessitating a cautious interpretation of the findings.

## 5. Conclusions

This study highlights the health and demographic factors associated with adverse birth outcomes among women with an IDD, underscoring the need for targeted strategies to improve maternal and infant health. The associations between Medicaid coverage, mental health disorders, gestational hypertension, and adverse birth outcomes suggest that addressing systemic barriers to care is essential for reducing disparities. Additionally, racial and ethnic differences in birth outcomes point to the need for equitable healthcare policies that ensure access to high-quality maternal care for all women with an IDD. By identifying and mitigating these risks, healthcare providers and policymakers can develop tailored interventions that promote healthier pregnancies and improve long-term outcomes for both mothers and infants in this vulnerable population.

## Figures and Tables

**Table 1:** Characteristics of pregnant mothers with intellectual or other developmental disabilities.

Variable	N = 5147
	%/Mean (SD)
Maternal age (SD) at birth	26.6 (5.8)
Maternal age category	
14–17 years old	118 (2.3%)
18–25 years old	2373 (46.1%)
26–30 years old	1374 (26.7%)
31–35 years old	860 (16.7%)
36–45 years old	422 (8.2%)
Maternal education	
High school graduate or above	4154 (80.7%)
No high school graduate	993 (19.3%)
Married at delivery	3711 (72.1%)
Race	
Non-Hispanic White	2903 (56.4%)
Hispanic	1997 (38.8%)
Non-Hispanic Others	247 (4.8%)
Medicaid coverage at delivery	649 (12.6%)
Smoking	484 (9.4%)
Number of prenatal care visits	
0–7 visits	865 (16.8%)
≥8 visits	4282 (83.2%)
Multigestational pregnancy *	144 (2.8%)
Maternal comorbid condition	
Gestational hypertension	360 (7.0%)
Gestational diabetes	221 (4.3%)
Mental illness **	546 (10.6%)
Seizure disorders	72 (1.4%)
Obesity	1421 (27.6%)

Note: * Multigestational pregnancy denotes a single pregnancy with more than one fetus. ** Mental Illness was defined as a diagnosis of depression, anxiety, schizophrenia, or bipolar.

**Table 2:** Percent of births with adverse birth outcomes (n = 5147).

Birth Outcomes	n	%
Preterm delivery	729	14.2
Low birth weight	609	11.8
Cesarean delivery	1274	24.8
Infant death within first year of life	87	1.7
Neonatal intensive care unit admission	249	4.8
Maternal pregnancy related infection	430	8.4

**Table 3:** Factors associated with adverse birth outcomes.

	**Preterm Birth**				**Low Birth Weight**				**Cesarean Delivery**			
	**OR**	***p*-Value**	**95% CI**		**OR**	***p*-Value**	**95% CI**		**OR**	***p*-Value**	**95% CI**	
Maternal age at delivery												
14–17 years old	1.11	0.72	0.63	1.95	0.84	0.61	0.43	1.64	0.76	0.32	0.44	1.31
18–25 years old	reference				reference				reference			
26–30 years old	0.87	0.22	0.70	1.09	0.98	0.87	0.78	1.24	1.52	<0.01	1.28	1.79
31–35 years old	1.04	0.73	0.82	1.34	0.87	0.31	0.66	1.14	1.77	<0.01	1.46	2.14
36–45 years old	1.26	0.14	0.93	1.71	1.52	0.01	1.11	2.09	2.40	<0.01	1.89	3.04
Maternal education												
Did not graduate from high school	0.95	0.65	0.75	1.19	0.93	0.54	0.72	1.19	1.03	0.78	0.85	1.24
Married at delivery	0.84	0.11	0.68	1.04	0.78	0.03	0.62	0.97	0.96	0.68	0.82	1.14
Race												
Non-Hispanic White	reference				reference				reference			
Hispanic	1.10	0.32	0.91	1.32	1.08	0.45	0.88	1.32	1.32	<0.01	1.15	1.53
Non-Hispanic Others	1.45	0.04	1.01	2.09	1.27	0.24	0.85	1.89	1.23	0.19	0.90	1.67
Multigestational pregnancy *	32.85	<0.01	21.43	50.37	22.82	<0.01	15.56	33.46	6.14	<0.01	4.27	8.83
Maternal Medicaid coverage	0.88	0.35	0.68	1.15	0.88	0.38	0.67	1.17	1.76	<0.01	1.45	2.13
Smoking	1.27	0.10	0.96	1.67	1.54	<0.01	1.16	2.05	1.20	0.11	0.96	1.51
Number of prenatal care visit												
0–7 visits	3.01	<0.01	2.47	3.66	2.92	<0.01	2.37	3.60	0.94	0.50	0.78	1.13
≥8 visits	reference				reference				reference			
Maternal comorbid condition												
Gestational hypertension	2.74	<0.01	2.09	3.60	3.23	<0.01	2.45	4.27	2.33	<0.01	1.85	2.94
Gestational diabetes	1.42	0.07	0.97	2.07	1.23	0.32	0.82	1.87	1.87	<0.01	1.40	2.50
Mental illness **	1.28	0.07	0.98	1.67	1.66	<0.01	1.27	2.17	1.16	0.16	0.94	1.43
Seizure	1.56	0.17	0.83	2.94	0.45	0.13	0.16	1.27	1.34	0.28	0.78	2.31
Obesity	0.76	0.01	0.63	0.93	0.80	0.04	0.65	0.99	1.45	<0.01	1.25	1.68
	**Infant Death in First Year**				**Mother’s Infection**				**NICU Admission**			
	**OR**	***p*-Value**	**95% CI**		**OR**	***p*-Value**	**95% CI**		**OR**	***p*-Value**	**95% CI**	
Maternal age at delivery												
14–17 years old	1.56	0.57	0.34	7.20	0.59	0.40	0.17	2.00	1.04	0.96	0.24	4.54
18–25 years old	reference				reference				reference			
26–30 years old	1.68	0.06	0.98	2.90	1.79	<0.01	1.37	2.34	1.84	<0.01	1.30	2.59
31–35 years old	1.61	0.14	0.86	3.01	2.09	<0.01	1.55	2.82	1.71	0.01	1.16	2.54
36–45 years old	2.11	0.05	1.02	4.38	2.29	<0.01	1.58	3.33	2.71	<0.01	1.73	4.24
Maternal education												
Did not graduate from high school	0.62	0.14	0.33	1.16	0.72	0.04	0.52	0.98	0.50	<0.01	0.32	0.77
Married at delivery	0.78	0.35	0.47	1.32	0.96	0.76	0.74	1.25	0.92	0.63	0.66	1.29
Race												
Non-Hispanic White	reference				reference				reference			
Hispanic	1.41	0.15	0.89	2.24	0.92	0.49	0.73	1.17	0.97	0.85	0.72	1.31
Non-Hispanic Others	1.42	0.44	0.58	3.47	1.33	0.19	0.87	2.03	0.94	0.82	0.53	1.66
Multigestational pregnancy *	2.71	0.02	1.14	6.40	0.72	0.37	0.35	1.47	7.20	<0.01	4.56	11.36
Maternal Medicaid coverage	1.62	0.10	0.91	2.88	8.07	<0.01	6.34	10.26	6.45	<0.01	4.74	8.78
Smoking	0.61	0.26	0.26	1.44	0.93	0.69	0.64	1.35	0.68	0.14	0.41	1.14
Number of prenatal care visit												
0–7 visits	3.79	<0.01	2.40	5.99	0.62	<0.01	0.44	0.86	1.68	<0.01	1.19	2.36
≥8 visits	reference				reference				reference			
Maternal comorbid condition												
Gestational hypertension	1.23	0.60	0.57	2.63	1.50	0.03	1.05	2.13	2.02	<0.01	1.35	3.02
Gestational diabetes	1.93	0.12	0.85	4.41	1.62	0.02	1.08	2.44	1.83	0.02	1.13	2.98
Mental illness **	0.87	0.71	0.43	1.77	1.98	<0.01	1.51	2.60	1.87	<0.01	1.33	2.62
Seizure	1.02	0.99	0.14	7.54	0.28	0.21	0.04	2.02	0.56	0.57	0.08	4.12
Obesity	0.87	0.58	0.53	1.43	0.76	0.04	0.59	0.99	0.65	0.01	0.47	0.91

Note: * Multigestational pregnancy denotes a single pregnancy with more than one fetus. ** Mental Illness was defined as a diagnosis of depression, anxiety, schizophrenia, or bipolar.

## Data Availability

Restrictions apply to the availability of these data. Data were obtained from the Utah Population Database and are available at https://uofuhealth.utah.edu/huntsman/utah-population-database and accessed 10 January 2022 with the permission of the Utah Department of Health and Human Services, the University of Utah Institutional Review Board, and the Utah Resource for Genetic and Epidemiologic Research (RGE).
